# IoT-Enabled Gait Assessment: The Next Step for Habitual Monitoring

**DOI:** 10.3390/s23084100

**Published:** 2023-04-19

**Authors:** Fraser Young, Rachel Mason, Rosie E. Morris, Samuel Stuart, Alan Godfrey

**Affiliations:** 1 Department of Computer and Information Sciences, Northumbria University, Newcastle-upon-Tyne NE1 8ST, UK; 2 Department of Health and Life Sciences, Northumbria University, Newcastle-upon-Tyne NE1 8ST, UK

**Keywords:** gait, internet of things, IMU, free-living assessment, telemedicine

## Abstract

Walking/gait quality is a useful clinical tool to assess general health and is now broadly described as the sixth vital sign. This has been mediated by advances in sensing technology, including instrumented walkways and three-dimensional motion capture. However, it is wearable technology innovation that has spawned the highest growth in instrumented gait assessment due to the capabilities for monitoring within and beyond the laboratory. Specifically, instrumented gait assessment with wearable inertial measurement units (IMUs) has provided more readily deployable devices for use in any environment. Contemporary IMU-based gait assessment research has shown evidence of the robust quantifying of important clinical gait outcomes in, e.g., neurological disorders to gather more insightful habitual data in the home and community, given the relatively low cost and portability of IMUs. The aim of this narrative review is to describe the ongoing research regarding the need to move gait assessment out of bespoke settings into habitual environments and to consider the shortcomings and inefficiencies that are common within the field. Accordingly, we broadly explore how the Internet of Things (IoT) could better enable routine gait assessment beyond bespoke settings. As IMU-based wearables and algorithms mature in their corroboration with alternate technologies, such as computer vision, edge computing, and pose estimation, the role of IoT communication will enable new opportunities for remote gait assessment.

## 1. Introduction

Human motion is fundamental to physiology [1] and perhaps its key measurable component is gait, where gait speed is now described as the sixth vital sign across many cohorts [2,3,4,5,6]. For example, gait irregularity/variability, asymmetry, and reduced velocity [7] are proving useful in the understanding of the underlying neurological mechanistic limitations reported in older adults (≥70 years) with dementia [8]. Alternatively, in what could be described as the opposite end of the physiological spectrum, the economy of movement in the running gait of younger adults (≈21 years) can be improved by changes in the foot strike to minimize ground contact time [9]. Accordingly, observing and analyzing specific gait characteristics can help to better understand a range of physiological outcomes across the life course for many purposes.

Typically, walking gait assessment has taken place under controlled observation by a trained professional (e.g., clinician, physiotherapist, or biomechanist), wherein an individual is prompted to perform a gait task with subjective observations informing subsequent decisions [10,11,12]. Naturally, such approaches are limited due to the subjective nature of visual assessment, often lacking validity and reproducibility [13,14]. Additionally, individuals may not portray habitual gait when under observation or may be subjected to scripted scenarios beyond their usual mobility patterns [15]. More recently, observational assessment has been augmented with digital technologies for a more rounded and insightful approach. Three-dimensional (3D) motion capture systems are considered the reference/gold standard, demonstrating suitable validity and reproducibility [16,17,18]. However, 3D motion tracking systems are mostly confined to a fixed (bespoke) location; they are expensive and require technical expertise and the precise attachment of many anatomical markers [19,20]. In contrast, wearable inertial measurement units (IMUs) have become preferrable as they usually have a much lower cost and are deployable in a range of environments [21,22]. Typically, IMUs contain a combination of accelerometer and/or gyroscope sensors to provide an understanding of acceleration and rotation, respectively. (Additionally, magnetometer IMUs (MIMUs) help to sense magnetic field strength and direction for a thorough examination of movement [23].) Powered by a range of specifically developed algorithms and machine learning instances [24], IMUs can measure a wide range of biomechanical properties, including gait phase estimation [25], point of impact, flexion angles, and asymmetry measures [26].

Wearable IMUs for gait analysis have been shown to be pragmatic tools for a wide range of areas, such as diagnosis and severity analysis within a range of neurological disorders (e.g., Alzheimer’s disease [27,28,29], Parkinson’s disease (PD) [29,30,31] and Huntington’s [32,33,34]); running gait analysis [35,36]; shoe recommendation [22]; and gait training through biofeedback [37] and as a component of sports-related concussion assessment [38]. However, the most notable developments are within neurological disorders, where the IMU quantification of variability and asymmetry gait characteristics is enabling a greater understanding of cognitive impairment [39] and fall risk [30]. Crucially, wearable IMU-based technology is often commercially available for immediate use outside of clinical/bespoke settings. Consequently, there is a paradigm shift towards assessing individuals in their usual environment to enable the understanding of habitual gait in a way which is more representative of an individual’s daily life [40,41,42].

The daily and routine collection of gait data would be of particular use in vulnerable cohorts, providing more clinically useful biomarkers to aid, e.g., fall risk prediction [43]. However, moving gait assessment beyond the clinic is fraught with several challenges. For example, wearable IMUs are typically reliant upon accurate anatomical placement and orientation [44]. Furthermore, under supervised gait assessment, wearable IMUs are typically mounted and removed for the extraction of data by a trained researcher/user. Naturally, this approach is less feasible in remote habitual environments and/or with those without routine familiarity, meaning that the data processing is often inefficient and rooted in manual processes.

A key enabler of the use of IMUs beyond the lab for remote gait assessment with automated processes is the Internet of Things (IoT). The IoT encapsulates the interconnectivity of devices, which often communicate with each other and with the cloud. With the prevalence of the IoT, new opportunities have arisen within remote healthcare through telemedicine, e.g., [45,46]. Particularly with regard to IMU-based gait assessment, the IoT could provide remote and more timely clinician monitoring (e.g., continuously informing on fall risk and/or the effect of interventions in the home). The use of the IoT would also reduce burdens (e.g., travel) and increase efficiency through transmission of data to the cloud, where the data can be processed and stored [47]. As a comparator, the field of blood glucose monitoring has readily available technologies with mobile connectivity via the interconnection of wearable and associated devices through use the of the IoT for remote clinical consultation [48]. As such, remote gait assessment could be streamlined through the use of the IoT, reducing the reliance on inefficient manual processes, which are fraught with difficulties within this domain.

Here, we perform a narrative review of the state of the current wearable gait assessment technologies, assessing for remote use. Specifically, our broad narrative approach helps to explore the role of the IoT as an enabler of remote gait assessment and to explore how the existing IoT-based technologies could be adapted to assist this contemporary area of research. Our narrative approach helps by including a range of interrelated technologies within and beyond wearables that must be considered and discussed within contemporary and IoT-enabled gait assessment.

## 2. Methods

The following review was conducted between September 2021 and January 2023. A search of electronic databases including PubMed, Scopus, Google Scholar, IEEEXplore, and Web of Science identified the relevant studies. No date range was used as an exclusion criterion in order to include a wide scope of the history and evolution of gait assessment technologies. The search terms included “gait”, “IoT”, “wearables”, “wearable sensors”, “IMU”, “computer vision”, “cybersecurity”, and “wireless sensing”. The literature search was limited exclusively to English language studies. Studies were excluded if they were outside of the search terms pertaining to gait assessment on humans in healthcare applications. For example, studies were commonly found regarding gait for robotic ambulation. Consequently, such studies were excluded as they did not fit into the narrative of this review.

## 3. Background: Digitizing Gait

Emphasis has generally been placed on the manual calculation of gait speed, which has been shown to be clinically useful when examining mortality and morbidity [49]. However, there is now significant appreciation that gait is no longer a routine task and is quite multifaceted with more to offer as the sixth vital sign [2]. Accordingly, digital technologies have been proposed as mechanisms to fully harness its (clinical) usefulness.

### 3.1. Considerations: Prescribing

It is important to note that each gait-affecting condition must be treated/examined in isolation as the technologies used to quantify specific gait characteristics can be interpreted differently to understand the underlying mechanistic problems [50]. To explain, some gait characteristics associated with different gait-affecting conditions are presented (a more comprehensive overview is provided elsewhere [51]).

- Hemiplegic and asymmetrical gait is prevalent in stroke survivors, wherein gait kinematics can differ between each leg/foot [51]. For example, stroke survivors exhibit decreased cadence, shorter stride length and stride time, with asymmetry between the lower extremities in many gait outcomes [52].
- PD gait will typically degrade over time, with incidence cohorts displaying lower step time, length, and velocity. As the disease progresses, affected individuals may be susceptible to wider gait asymmetry and bouts of freezing of gait (FOG) [53]. Additionally, a weakened pace and rhythm can be experienced within Parkinsonian gait [54].
- An increase in gait variability, as well as fluctuations in stride length and speed can be indicative of reduced cardiovascular health, often contributing to an increased risk of future health conditions [55].
- Ground contact time and the total step time show a significant increase when orthotics (shoe inserts) are used, suggesting that the latter do not interfere with running techniques and could be used as a preventive injury tool during running [56].

**Figure 1:**
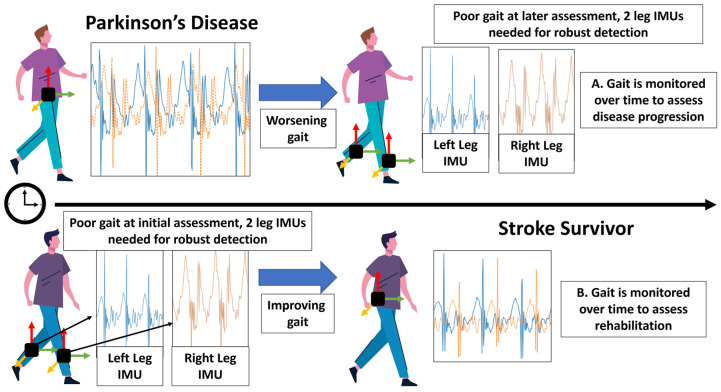
In prescribing IMUs for gait assessment, one size may not fit all. Considerations of placement selection between PD and stroke, where different colored signals represent examples of different sensing axes of acceleration. Top: use of a single IMU on the lower back is useful in early PD to detect, e.g., gait variability, but a single IMU may not be fit for purpose as gait worsens. Accordingly, use of two IMUs on the legs may be more appropriate to detect slower, shuffling gait often seen in advanced PD. Bottom: a contrasting example, IMUs placed on the legs of a recent stroke survivor may be more optimal for gait assessment compared to the lower back. As gait for this person improves though a rehabilitation program, use of a single IMU may be more optimal and pragmatic.

Consequently, given the differing range of gait features and applications, it is paramount that the most suitable IMU/MIMU configuration (accelerometer, gyroscope, and magnetometer) and attachment location(s) are selected to ensure a complete and robust quantification of the gait characteristics of specific cohorts. Furthermore, those choices will inform the selection and/or development of arising software/algorithm methodologies. For example, in Figure 1, an incident PD cohort may have a gait pattern that is robustly quantified with the use of a single IMU on the lower back, giving valid (robust) and insightful gait variability outcomes [57]. Alternatively, a recent stroke survivor may have an extremely impaired gait; so, it may be more suitable to have, e.g., two IMUs on each leg [58]. Accordingly, it is important to note that one size does not fit all and that gait assessment with the associated digital technologies such as IMUs may need to be considered within the context of a prescription-based model.

### 3.2. Instrumenting Gait: Approaches and Considerations

#### 3.2.1. Inertial Measurement Units

In their typical use case, IMUs are worn at precise anatomical locations, depending upon the research question [44]; e.g., what is the most sensitive gait outcome for the patient group to examine the disease type and/or the effect of an intervention? Again, this is related to the topic of prescription and to ensuring that the best IMU configuration is employed to address the research need. Accordingly, quantification of the desired gait outcome is dependent on the selection of the correct IMU configuration.

The selection of the correct IMU can inform the assessment type and length. IMU-based devices include several commercial options, ranging from the low-cost and open source to the more expensive and closed source (i.e., black box). Each option has advantages and disadvantages depending on the research needs, but readers are directed to other work to explore some available options for supervised and free-living (low- to high-resolution) gait [59]. Listing a full range of IMU devices and their applications is outside of the scope of this narrative review. However, extensive reviews of commercially available IMUs and their respective applications can be found elsewhere, e.g., [26,35,60,61].

Often, depending on the desired gait outcomes, it may be crucial to consider a multi-sensor setup. For example, Sabatini et al. [62] provided an estimation of stride length using a single foot-mounted IMU. Although the approach demonstrated relative accuracy, the authors discuss how a multi-sensor configuration may improve the accuracy by providing data on multiple anatomical locations. This notion extends to joint kinematics, which can inform additional clinical evaluation. For example, Niswander et al. [63] found that the optimal measurement of the joint angles of the leg (i.e., hip, knee and ankle) requires multiple IMUs placed upon the sacrum (base of the lumbar vertebrae), lower anterior thigh, and lower lateral shank and upon the heel. As such, for a full suite of gait outcomes, a multi-sensor configuration may be required. Despite this, the pragmatics of a multi-sensor configuration should be considered as such a setup may limit, e.g., free-living assessments through the complex attachment protocols. Additionally, multiple sensors will naturally lead to higher costs, which may inhibit the feasibility of some studies.

Upon IMU(s) selection, it is important to consider the sampling rate of the sensor. There is no standardized rule on the sample rate selection of IMUs in gait analysis, as the sample rate selection can vary depending on the desired outcomes and selected sensor. However, some guidelines can be extracted from the research domain, which may inform selection. For example, Klieme et al. [64] found that within walking gait feature extraction, there was a negligible improvement in feature extraction performance between the 25 Hz (low-frequency) and 400 Hz (high-frequency) inertial streams. Despite this, a large proportion of studies opt for a sampling rate in the range of 40–100 Hz (avg. ~50 Hz) [65]. Indeed, a higher sampling frequency (e.g., 400 Hz) may be the most accurate for intricate gait feature extraction in certain applications [66]. However, the sample rate selection may be better considered as a tradeoff between resolution and test length. For example, a long-term gait assessment (e.g., 1 week) may benefit from a lower frequency to maximize battery life and minimize file size, whereas short-term assessments (e.g., 2 min walking test) could warrant a higher resolution, where battery life and file size are of less concern. Additionally, when utilizing a multi-sensor configuration, it becomes increasingly important to optimize the sampling rate, as file size will proportionally scale with the number of sensors, which may lead to inefficiencies in the data analysis.

#### 3.2.2. Considerations and Limitations

IMU-based gait assessment generally relies upon a suite of algorithms and/or machine learning instances to extract useful gait outcomes. However, to date a significant portion of algorithm developments have relied upon research-grade, closed-source software suites and packages such as MATLAB**^®^** for execution, which still require some manual intervention, e.g., [67,68,69,70,71,72]. This potentially limits use in low-resource settings, i.e., it cannot be deployed outside of the closed-source software and the need for technical knowledge. In response, there has been a recent emphasis to move gait analysis from, e.g., MATLAB**^®^** to open-source mechanisms, where Python has been most prominent. For example, the GaitPy package [73] provides an open-source framework for the gait classification, gait feature extraction, and data visualization of lower-back-mounted IMU data, implementing common algorithms by McCamley et al. [71] and Ziljstra and Hof [74]. By providing an open-source platform to perform gait analysis based on wearable data, the field can become more accessible to a wider range of researchers who do not have access to the tailored software.

Longitudinal IMU deployment for remote gait assessment can last up to, e.g., 7 days [75] to quantify trends during habitual patterns of activity/behavior. As IMUs are reliant on precise anatomical placement and orientation, the dependency on the participant to attach and remove the IMU (e.g., when bathing) may lead to attachment error [76]. This consideration can reduce the effectiveness of deployment when trying to capture robust gait outcomes. As described, the choice of gait outcome (IMU configuration, sampling frequency, and wear location) may impact battery and memory life, potentially limiting long-term recording [59]. Accordingly, the choice of (commercial) IMU has several important logistical considerations. For example, although the AX6 (Axivity) has a lower cost and can continuously record inertial data for many days, it is without proprietary software to routinely process synchronized IMU data for gait assessment and must be deployed with the participant and collected after many days. Alternatively, at a higher cost, the APDM Opal with Mobility Lab software [77] can provide a structured gait assessment with synchronized IMUs but requires frequent recharging (primarily due to Bluetooth functionality). Equally, both systems require continued researcher intervention to process the large amount of data and the subsequent generation of gait characteristics. For example, IMU devices will typically be attached and removed immediately before and after a gait task within a supervised setting. Subsequently, a manual data extraction and analysis process may occur, which equates to an inefficient process [78,79,80]. This is often exacerbated when using multiple IMUs during a collection of a large amount of data (i.e., many participants). Furthermore, depending on the gait outcomes required to address the research needs, a large volume of IMU data could negatively impact download and analysis timelines. Hence, there can be an identifiable bottleneck experienced in many settings when undertaking manual IMU data extraction. As such, providing mechanisms to remove the reliance on researcher intervention for IMU-based gait assessment could streamline the domain.

#### 3.2.3. Overcoming Obstacles

Some commercial IMU technologies enable automated processes and near-real-time monitoring by streaming the inertial data, but these advanced capabilities are unaffordable and/or too technically demanding for routine deployment. Their use remains limited to controlled/bespoke settings, and/or they are generally not fit for purpose in a home. There is a need to enable remote gait assessment through existing technologies that are readily accessible and scalable. For example, Leite et al. [81] developed a gait rehabilitation monitor with a commercial IMU which streamed data to a smartphone application/app. In that study, pre-processing took place before classification of the gait characteristics and was performed in the cloud, removing the time-consuming need to return the IMU to a researcher for manual data extraction and analysis. Interestingly, that study showcased an alternative and potentially more economical approach (i.e., lower cost) through integration with the ubiquitous smartphone.

#### 3.2.4. Smartphones

The ubiquity of smartphones in modern life [82] has significantly contributed to digital health by providing accessible technology with wireless communication and embedded inertial sensors and camera(s) [83,84,85]. The inclusion of those sensing modalities has enabled smartphones to provide a range of physiological measurement and data entry/collection capabilities, via apps, in an IoT context [86]. Peripheral wearable technologies broaden data collection with notable examples, including popular commercial devices such as Apple Watch, FitBit, and Garmin. In practice, the smartphone acts as a central communication hub for external devices by sensing data, generating visualization of the data, and communicating the data to a central database (in the cloud). Gait assessment and the need to move it beyond the clinic could benefit from the smartphone’s unobtrusive, deployable nature [87] that may be more accepted by the end user (e.g., older adult and their own phone) [88]. Indeed, the use of a person’s own phone means s/he has familiarity with the technology and an already deployed (gait assessment) infrastructure. As such, smartphones could facilitate remote, long-term assessments that were previously unattainable.

##### Limitations

Although smartphones may provide a current and pragmatic mechanism for remote assessment, the technologies are fraught with limitations that are important to consider. Firstly, adhering to healthcare data security standards when using software as a medical device (SaMD) can be challenging given the ever-changing range of hardware and software associated with healthcare-based smartphone applications [89]. As such, regulatory bodies are often cautious about approving smartphone-based healthcare devices. In addition, the ‘always online’ nature of the IoT-based smartphone presents additional challenges for remote assessment considering the increased security risk. For example, smartphone users’ awareness and use of security best practices is limited, with many users disregarding guidance such as avoiding public networks and accessing insecure networks without relevant protection (e.g., virtual private network, VPN) [90], potentially increasing the risk of data theft. Given the older adult demographic primarily associated with remote gait assessment for fall risk, this issue could be exacerbated, given the demographics of relatively low familiarity with the technology in comparison to younger generations and their use of the same type of technology for health and fitness [91]. Consequently, before use as remote assessment devices, particularly with regard to older adult health, security concerns will require significant consideration, including educating affected users or requiring, e.g., VPN for use.

## 4. IoT as an Enabler for Remote Assessment

IoT communication between sensing modalities and external machines has provided complex analysis methods in near-real-time to the end user [92,93,94]. For example, the IoT has produced remote healthcare applications for the home-monitoring of vulnerable patients [47], cardiovascular assessment [95], quality-of-life measurement [96], and near-real-time disease classification [97,98]. However, there has been a dearth of technical advances to provide gait outcomes due to the clinical and technical considerations [96]. To consider how gait could be remotely assessed with the aid of the IoT, it is necessary to describe the fundamental approaches. Firstly, a sensing modality may need to:

(i) Gather or stream data to an edge computing device [99];
(ii) Relay data to a cloud instance [81];
(iii) Process data via a series of classification models and/or algorithms (e.g., deep learning instances) for desired gait outcomes [96,100].

**Figure 2:**
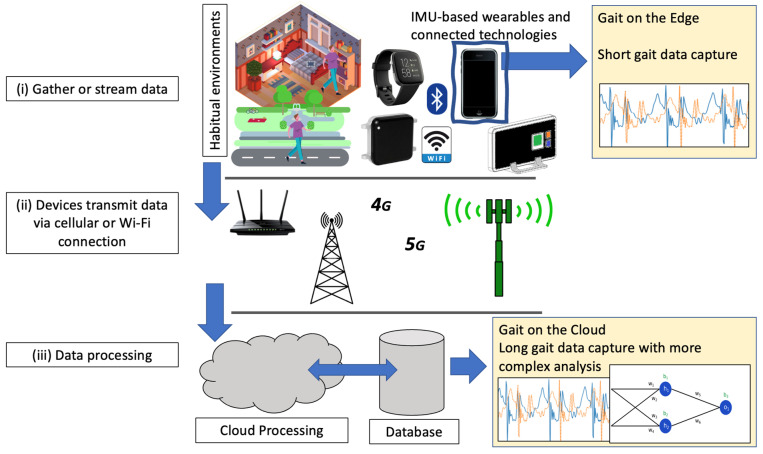
Considerations of remotely assessing gait within an IoT context, demonstrating (i) a collection of IoT-enabled wearable and deployable devices streaming to an edge computing instance, e.g., smartphone; (ii) data being transmitted through wireless infrastructure from edge computing devices; and (iii) receiving data within a cloud instance for processing and storage.

The following sections discuss those areas and explore the need for future development; see Figure 2.

### 4.1. Data Capture: Commercial Ubiquity

As a combination of data capture, streaming, and storage mechanisms, smart devices (e.g., smartphones) could provide direct apparatuses for remote assessment. Early work [101] utilized an iPod with an embedded tri-axial accelerometer and tri-axial gyroscope mounted on the lower back to implement near-real-time gait assessment; this shows how IMU data within smart devices can be harnessed. Although the referenced study made use of an iPod, its functionality forms the basis of current smartphones with the added benefit of routine IoT-based wireless data transmission and other ad hoc apps that could use IMU and/or other data, e.g., a camera. Utilizing the existing commercial and readily available technologies such as smartphones could be a realistic mechanism in the process of remote gait assessment as this utilization can overcome the most challenging barriers of (i) immediate technology deployment and (ii) the distribution of high costs [99]. Specifically, with the ubiquity of smartphones, a large proportion of the infrastructure and associated costs has already been absorbed by the public, rather than bespoke technology provided by a central agency.

The use of existing IoT-based smartphone infrastructures, including apps, could also enable the inclusion of other peripheral commercial technologies that are already integrated. Specifically, the use of Bluetooth-enabled wearables could augment more tailored assessments at different locations of the body. For example, a bespoke, Bluetooth-enabled, embedded IMU-based smartwatch [86] or smart sock [22] could transmit inertial data from the wrist and/or foot/feet to a smartphone for processing to provide more tailored outcomes. However, with relatively low-energy devices such as smartphones exhibiting low computational power, their use as (big) data processing apparatus may be limited for complex assessments that rely on high computational power, such as the use of artificial intelligence [102]. Rather, the use of a smartphone as the conjugate device (Figure 2i) for data capture via Bluetooth from a peripheral inertial sensing wearable and onward transmission to a higher-powered (i.e., more computationally equipped) cloud instance may be the optimal approach. Subsequent storage and/or further processing of gathered data [22,86,87] would produce specific gait outcomes. For example, an IoT-enabled, wearable, foot-mounted IMU [22] has been shown to successfully transmit 1 min bouts of running data to an iOS/Android smartphone before the latter packages the data and communicates with a cloud-based feature extraction and deep learning instance. This minimizes the computational power required for local analysis on the smartphone.

### 4.2. Local Networks: Data Handling

Modern high-capacity wireless connectivity through Wi-Fi or cellular networks (e.g., GSM, 4G, 5G) could enable ubiquitous functionality via smartphones to routinely inform high-resolution gait assessments within the IoT. However, this brings challenges where wireless infrastructures may be limited (e.g., under-developed regions, low-resource settings). More broadly, the global deployment of IoT systems for telemedicine can be challenging because at home the Wi-Fi uptake in older populations can be limited [103] despite the general adoption of such technologies in that age group in developed countries [104]. Yet, several governments [105,106,107] have committed to developing digital infrastructure as part of the effort for widespread 5G access. In particular, the evolution of cellular communication to 5G and beyond will increase the efficiency of data streams by providing higher bandwidth, speed, and greater accessibility [108]. This may provide an opportunity for streaming gait-ready data to the cloud.

Improved and accessible cellular networks could enable, e.g., multimodal gait assessment in any environment (i.e., outside of the lab and into the home and beyond; see Figure 2i). For example, Celik et al. [109] performed a multi-sensing (IMU and electromyography) assessment in varying terrains and environments to more comprehensively understand gait in uncontrolled settings which contain naturally occurring obstacles that may be poorly replicated in a lab. The approach utilized strenuous manual extraction and analysis of multi-sensor data. As a solution, the approach could utilize a smartphone for data collection from the sensing modalities, wherein the data are packaged and streamed to a cloud instance via a smartphone to provide near-real-time gait outcomes, as previously demonstrated [22]. However, technical, and pragmatic challenges must be considered when adopting a data streaming approach, including how many sensing modalities poll at high frequencies that may be unsuitable for consistent streaming. For example, a 100 Hz polling rate with tri-axial accelerometer and tri-axial gyroscope [59] provides up to 600 data points/second. Although this may be suitable for monitoring short walking bouts [22], the approach will be almost completely unfeasible for the long-term data capture sessions that are typical in remote assessment [110] due to the maximum data usage concerns, battery life, and associated costs [96].

A comparison of different cloud platforms for streaming and storing IoT-collected data was recently presented [96]. Generally, cloud platforms are inefficient for the streaming and storage of large amounts of sensor data due to ‘pay-as-you-go’ models based upon usage. However, for low-frequency intervals such platforms excel, indicating a suitability of cloud platforms for edge computing-based IoT projects, where gait outcomes are quantified locally (e.g., on a smartphone) before being pushed to the cloud, rather than large raw (sample level) data streams.

### 4.3. Edge Computing: High Frequency Data and Streaming

An edge computing approach to capture high frequency gait data and streaming is perhaps the most effective use of smartphone computation and data transmission limitations, within the realm of existing IoT infrastructures. One proposal may be a four-step architecture, such as:

1. Sensing modalities with an option of multimodality: IMU-based smartphone with or without an external wearable device. Data captured by a smartphone and/or transmitted via Bluetooth to the smartphone, wherein the data are stored [22,101,111].
2. Computationally inexpensive analyses are performed on-device to extract key features, regions of interest, and bouts of gait [101]. For example, during free-living assessment there will be periods of sedentary behavior (e.g., the person is sitting still or sleeping). As such, identifying regions of interest in the signal is paramount to provide the most relevant data for analysis. Pre-existing and validated approaches to identify gait in free-living scenarios [79,112] could be adapted for use on a smartphone due to relatively uncomplex computations (i.e., no use of deep learning). By identifying regions of interest within a high frequency signal before transmission, bandwidth/costs would be reduced by the sending of shorter signals.
3. Regions of interest are transmitted to a cloud instance for a range of analyses and long-term data storage [22,113,114]. Gait algorithms can be controlled centrally and altered as required.
4. As seen in other domains such as autonomous vehicles [115], full datasets (i.e., not pre-processed on-device to define regions of interest) could be uploaded to a cloud instance for storage at less-regular intervals where data bandwidth was not of concern, e.g., if a Wi-Fi signal was available.

By utilizing a smartphone as an edge device with pre-processing capabilities, the field of remote gait assessment can be improved by streamlining data capture, lowering data usage, and minimizing costs [116].

### 4.4. Data on the Cloud: Storage and Security

Gait assessment naturally promotes heterogeneous data with widely varying sensing modalities (e.g., IMU in different locations and polling frequencies), informing gait assessments across a spectrum [117,118,119,120,121], which may require highly specific and tailored approaches; see Figure 1. Consequently, the storage of heterogeneous data presents an array of technical considerations when discussed in the context of gait assessment and cloud computing. In particular, there may be no current one-size-fits-all approach to selecting data storage and security for a broad-ranging data-driven domain such as gait assessment.

#### 4.4.1. Data Storage

The majority of IoT-based healthcare developments lack data interoperability and contain fragmented data, which causes logistical issues in understanding healthcare data [122]. Indeed, this notion is exacerbated within the field of gait assessment, especially given the prevalence of high-frequency data (e.g., 100 Hz [59]) and the kinds of self-reported symptoms [123], as well as clinician and observational notes [14]. NoSQL database structures have been explored in healthcare due to the heterogeneous form of monitoring devices and methods used in that sector [124]. Although a NoSQL database may have an intrinsic use for managing fragmented gait data, not all NoSQL database technologies perform equally within an IoT context. To assess their respective suitability in different execution scenarios, the performances of major NoSQL distributions have been examined [125]. In that study, column family-based databases such as Cassandra and HBase were found to perform best under read/update scenarios of high-frequency data, whereas document and key-value in-memory databases such as MongoDB, Redis, Memcached, OrientDB, and Voldemort are optimal in reading operations. Consequently, when designing an IoT-enabled gait assessment system, the database type should be considered with regard to the frequency of data input and the number of reads. For example, in use cases where frequent data transmission occurs (e.g., streaming of multiple IMU data stream), write-optimized distributions such as Cassandra should be considered. Alternatively, for primarily read-focused operations (e.g., accessing raw data streams of long-term data capture sessions), the likes of MongoDB may provide a more optimal performance.

#### 4.4.2. Data Security

As with many internet-enabled technologies, concerns regarding the long-term security of healthcare data [126] and the current lack of a standardized framework for the storage and access of data [96] have contributed to the current under-adoption of IoT devices in healthcare outside of a research context. Despite this, it is widely accepted that the endorsement of IoT-enabled wearables will be seen in the near future due to the streamlining and cost-reducing nature of the technology [127]. As such, it is paramount that data security standards are considered within the field of IoT-enabled gait assessment. Recently, there has been a significant interest in developing and adopting a standardized data security standard for healthcare data with regard to the IoT, e.g., [128,129,130]. Here, key concerns from the field are briefly summarized and considered.

##### Identity and Anonymity

Providing anonymous mechanisms for data storage and authentication is paramount to the uptake of and user confidence in wider IoT-enabled healthcare assessments [131]. In particular, concerns arise regarding the sensitivity of biometric and healthcare data and the impact that data leaks may have on privacy and identifiability. This notion extends to the field of IoT-enabled gait assessment; as each individual exhibits a unique gait pattern [132], this warrants the gait’s use as a biometric feature, and as such, it can be used to identify and distinguish individuals [133]. Consequently, secure methods must be considered when developing an IoT-enabled gait assessment tool. It is well established that utilizing a public and private key-pair approach to encrypt/decrypt data between patients and healthcare professionals can assist in enabling anonymity, i.e., [134,135,136], and it should be implemented as a priority in healthcare systems. However, key-based approaches can often rely on the reliability and trustworthiness of, e.g., cloud providers [134]. As such, it may be necessary to explore alternate or supplementary mechanisms for gait data anonymity. For example, Delgado-Santos et al. [137] proposed an IMU-based gait signal anonymity tool utilizing unsupervised learning with autoencoders to remove sensitive data from signals. Such an approach could feasibly be adapted for use in gait data storage, wherein a prospective attacker would need to not only compromise public/private key pairs but also obtain a specific auto-encoder to meaningfully understand the signals.

##### Subjectivity and Resistance to Malicious Attacks

As with all healthcare and identifiable data, a suitable resistance to malicious intent is essential before use in a commercial or research-grade product. Recently, there has been considerable discussion regarding e-healthcare, the IoT, and security standards. In particular, there is a significant threat regarding compromising the integrity of medical hardware and devices, with many existing e-health systems relying on out-of-date software/hardware stacks that have become obsolete since their initial deployment [138]. As a solution, the study recommends making use of cloud computing services with tailored solutions for high-sensitivity data, while providing mechanisms for data loss and hardware redundancy. Observing the current gold standard within healthcare data storage, Azure’s Health Data Services [139] provides a secure means of real-time healthcare data storage and transmission. In addition, the Azure platform adheres to many global and regional security standards, including the ISO 27081 global standard and GDPR EU standards, ensuring the utmost possible security.

### 4.5. Data Processing in the Cloud

In rapidly evolving fields such as remote gait assessment, sensing modalities generally require a series of complex analysis and processing steps. As an example, Bikias et al. [140] proposed a local (offline) complex deep learning method for identifying freezing of gait (FOG) in PD patients from an IMU, wherein wrist-mounted tri-axial accelerometer and tri-axial gyroscope data informed a convolutional neural network (CNN)-based architecture to analyze the data. The use of the cloud could enable a scalable approach to FOG data processing and analytics through providing a modular system architecture [141]. Furthermore, utilizing the cloud for gait data processing could also enable a wider range of complex assessments. Recently, the use of artificial intelligence and deep learning has seen significant interest with regard to the gait, e.g., [95,142,143,144]; subsequently, a wide array of outcomes have been quantified. However, due to artificial intelligence often requiring significant computational power, the approach may not be suitable for operation on consumer-grade hardware (e.g., a low-powered smartphone). As such, by utilizing the cloud for data processing, should a particular algorithm require significant computational complexity (e.g., deep learning), a cloud infrastructure can scale by deploying extra resources at the point of execution [145], removing the reliance on low-powered consumer hardware.

To date, several studies have proposed cloud-enabled IMU gait assessment, including studies considering neurological conditions [146], gait rehabilitation [147,148], and sporting applications [22,25]. By performing analysis with the cloud as opposed to local processing, several key benefits are established. For example, Yang et al. [148] utilized a modular approach to remote gait assessment by proposing three key components; the ‘Perception Module’, ‘Gait Analysis Module’, and ‘Display Module’, handling data capture/streaming, data processing/analysis, and data interpretation, respectively. By providing a modular approach, it is feasible to interchange components of a cloud-based system. For example, the proposed gait analysis module quantifies gait phases using temporal gait features. As research advances, fully assessing, e.g., older adult health may require a wider range of outcomes [149] which have yet to be quantified/described and shown as useful. As such, the deployment of new/improved algorithms to a cloud instance could expand the functionality of existing approaches, reducing the previous logistical and pragmatic shortcomings, such as having to, e.g., update a smartphone application, which could be taxing for older adults in unassisted remote contexts [150]. This notion has already been applied in consumer grade cloud-enabled running gait assessment, wherein, firstly, a modular IoT infrastructure is defined based upon smartphone data capture and a cloud processing unit [22], and an initial set of gait outcomes are selected for extraction. With regard to the initial set of gait outcomes, as new methods were validated, such as temporal feature extraction [25] and increased resolution [151], they were adapted to a pre-existing cloud infrastructure, demonstrating the utility of a ‘plug-and-play’ cloud-enabled approach to remote gait assessment.

Interestingly, the use of the cloud in an IoT-enabled environment may solve shortcomings associated with sensing modalities through enabling a multi-modal approach. A common concern within IMU-based gait assessment is the lack of context the sensors provide within free-living scenarios [117]. For example, an IMU may identify a highly irregular bout of gait in, e.g., a neurological patient; however, it cannot define whether the irregularity was intrinsic (caused by the neurological condition) or extrinsic (e.g., maneuvering around a complex environment) [152]. As such, there is a need to provide context within IMU gait assessment. This could be achieved through the combination of technologies (e.g., computer vision) communicating via an IoT and cloud infrastructure, solving the respective shortcomings of sensing modalities.

## 5. IoT-Enabled Multi-Modal Remote Gait Assessment

A combination of sensing modalities may be necessary to augment an effective remote gait assessment to overcome the shortcomings within the respective approaches (e.g., IMU). Through utilizing the IoT, sensing modalities could communicate in an ensemble and thus provide a wider range and better-informed assessment.

### 5.1. Computer Vision and Gait Assessment

Computer vision (CV) is generally considered in two categories: 2D (a regular camera) and 3D (use of camera in conjunction with technologies such as infrared, e.g., Microsoft Kinect) [153]. CV has shown utility in providing insight into a variety of healthcare applications such as remote monitoring of vulnerable cohorts [154], fall detection [155,156], and running [157]. Typically, 3D-based CV is considered the gold standard in person identification due to its understanding of depth perception [158]. However, the gold standard approach currently incurs a high cost and the need for professional supervision [159], which limits its utility outside of controlled environments. Consequently, 2D has become a springboard to provide a pragmatic approach.

Recently, the 2D-based CV has been shown to be extremely effective in the understanding of environments, identifying environmental hazards [160] and context [161]. CV has also excelled in human activity recognition (HAR) [162], providing an understanding of the actions of a human in their habitual environment. Generally, such CV applications rely upon artificial intelligence to perform their respective tasks. For example, utilizing mask region-based CNN (RCNN), Long et al. [163] identified and segmented obstacles within habitual environments from a video stream, providing environmental understanding to those with limited sight, demonstrating the efficacy of contextual CV. Additionally, HAR has become increasingly valid given the rise of accurate pose estimation techniques such as OpenPose [164], a spatiotemporal, anatomical key point identification framework based upon part-affinity fields. Through utilizing pose estimation for HAR purposes, we can begin to understand the relevant actions a human may exhibit within habitual environments that could be clinically relevant within gait assessment, including postural transitions [165], gait initiation [166], and fall detection [167]. Furthermore, pose estimation can provide useful gait outcomes without the need for markers or wearable technology; it excels at leg flexion and extension angles, as well as postural (e.g., spinal alignment in relation to the body) outcomes that can be used for rehabilitation purposes [168].

Understanding the environment and how an individual navigates it is paramount in providing a free-living gait assessment. In a typical lab-based assessment, patients will walk in obstruction-free environments over, e.g., an instrumented walkway for 10 m to assess gait parameters relevant to their neurological condition [169]; however, such tests are not indicative of how a patient may move within their habitual environment. For example, a patient could be exhibiting slow/irregular gait due to, e.g., walking or running with another person, which an IMU could not accurately distinguish from irregular gait caused by an intrinsic factor. Consequently, CV could be used in conjunction with an IMU to overcome the shortcomings of each and provide a harmonious ability for free-living gait assessment. A hypothetical approach could utilize a smartphone as a central unit in an IoT system, where a CV-based application could provide contextual information (environment and obstacles [170,171]), while a wireless-enabled IMU streams the inertial data [99] for intricate gait outcome extraction.

#### Considerations

Naturally, the use of CV and the IoT within healthcare applications creates a wealth of privacy, legal, and ethical issues that have been extensively researched within the domain [127,131,172,173,174,175] and would be out of the scope of this narrative review. However, key findings from the field are summarized here. There is a need for anonymous, unidentifiable CV standards and methodologies to be developed before any meaningful progression in the pragmatic use of CV in habitual environments. This discussion informs the wider context of CV [176], evaluating the relevance, storage time, lawful processing, and accuracy of ethical CV systems [177]. Although useful for general surveillance in large environments (e.g., CCTV), CV’s utility in habitual environments requires significantly more complex considerations, with the inclusion of highly personal events in habitual life (e.g., children in view or other sensitive material such as bank statements). Consequently, when designing a CV system, steps should be taken to alleviate such concerns. Techniques such as image obfuscation are often deployed within the wider context of CV privacy, including image blurring, blocking (placing obstructions in front of sensitive content), or image segmentation (only following the interested individual) [178,179], obscuring video streams against any malicious intent. However, particularly within remote gait assessment, understanding the extrinsic factors affecting an individual’s movement is crucial to providing a context for additional assessments [180]. As such, obscuring potentially large portions of video streams may hinder assessment.

As a solution, adopting a privacy framework such as privacy by design [181] may improve security and the ethical shortcomings associated with habitual CV; within this framework, systems should function only within their designed constraints, with no deviation from the intended purpose. For example, Moore et al. proposed a CV tool to assist in contextualizing IMU data with a head-mounted video camera (smart glasses) [152]. In a related but separate case study, autonomous CV classified walking terrain to inform gait outcomes obtained from IMU signals [180]. Crucially, the CV application does not store images for further viewing; rather, it only stores the classified outcomes (e.g., an individual is walking upstairs) and their respective timestamps to provide context to blind IMU data streams. Further adapting this approach to include a wider range of HAR outcomes and environmental outcomes could therefore alleviate privacy concerns through autonomous classification and stored outcomes.

### 5.2. A Proposed Approach: The Complementarity of IMU and Computer Vision

**Figure 3:**
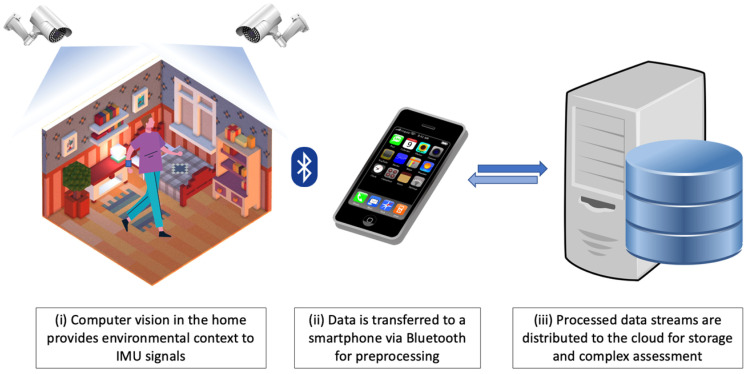
A proposed infrastructure for multi-modal gait assessment in habitual environments. Here, (i) is informed by a computer vision-based system to provide environmental context; a low-cost wearable IMU streams inertial data to a (ii) smartphone, where preprocessing takes place before transmission to (iii) the cloud for storage and further analysis/assessment.

Here, a proposed approach utilizing the IMU and computer vision is described; it involves communicating with a smartphone as the central unit, managing communication between devices, and transmitting data to the cloud for storage and additional processing; see Figure 3. Using a multi-modal approach, assessments could be tailored depending upon the conditions, the gait outcomes of interest, and the recommendations.

#### 5.2.1. Wireless-Enabled IMU

A wireless-enabled IMU with Bluetooth communication can be selected and placed in a relevant location based upon the gait features of interest. For example, a wireless-enabled IMU mounted to the lower back could be worn to measure lower-limb kinematics and estimate gait asymmetry between the left and right extremities [77] through the use of an algorithm or machine learning instance such as that proposed by Lim et al. [182]. Additionally, a consumer-grade wearable device (e.g., smartwatch) could be deployed to measure additional outcomes of interest such as heart rate, which is a holistic approach useful in different cohorts [183,184].

#### 5.2.2. Smartphone as a Central Communication Node

Operating as a ‘central node’ within the IoT system, a smartphone could be responsible for data capture, sensing, and transmission at a relatively low cost. In particular, the smartphone could receive inertial data from a wearable IMU via Bluetooth and perform light processing on the data (such as gait identification [185]) to minimize the need for large data packets to be consistently uploaded to a cloud instance. Additionally, sensors commonly found within a smartphone may also be utilized. For example, GPS data could be intermittently read to provide additional context (outdoor vs. indoor). This could be especially useful in identifying terrain type, which would have natural effects on a patient’s gait (e.g., walking on flat ground will differ vastly from decline walking) [109].

The use of a smartphone creates avenues for real-time gait feedback, based upon extracted gait outcomes. For example, [186] demonstrates the efficacy of real-time assessments through communication between wearable technology and a smartphone platform in identifying and extracting temporal gait outcomes on-device. By adapting such an approach, the smartphone could be used to assist in remote and/or automated rehabilitation sessions, allowing patients to receive gait prompts or useful metrics that could inform, e.g., gait cueing to aid symmetrical gait in PD [187].

#### 5.2.3. Environmental Context

To provide an environmental context to remote gait assessments, a camera stream or pictures could be deployed either (i) within the home [188] or (ii) as wearable/integrated in a smartphone [189] to identify a range of information surrounding the patient. Using camera data would alleviate the shortcomings found in solely IMU-based assessment, providing context to clinicians as to what the patient was doing and where the patient was at specific moments in time. As such, features extracted from inertial sensors could be distinguished as being either intrinsic (caused by neurological impairment) or extrinsic (e.g., avoiding an obstacle) factors, which may help refine remote gait outcomes. By wirelessly transmitting contextual data to near-real-time and unsupervised remote monitoring may be improved by removing the false positives obtained from extrinsic factors.

#### 5.2.4. Data Transmission, Storage, and Cloud Infrastructure

The heterogeneity of multi-modal gait assessment data permits the use of NoSQL data storage due to scalability, dynamic data structures, and cost [190] over SQL-based counterparts. When applied to potentially high frequency data associated with gait assessment sensing modalities, consistent transmission may prove unsuitable due to the transmission costs associated with hosting platforms [96].

Considering the costs associated with high-resolution data transfer in common cloud providers [96], the use of edge computing may indicate a higher level of suitability for enabling remote gait assessment within the IoT [116]. A smartphone would enable assessments in a wide variety of environments, transmitting infrequent data packets over cellular data (e.g., 5G), while waiting for Wi-Fi connection to transmit full data streams for research and clinical purposes.

## 6. Conclusions

Moving gait assessment into habitual environments is paramount to take the next step into routine monitoring across a wide spectrum of research domains. Currently, there are several shortcomings associated with the field, such as heterogeneous data capture methods, unstandardized data storage standards, and a vast range of approaches, depending upon the condition.

Here, the current gold standard in remote gait assessment is described, with inertial measurement units seeing primary use due to their relatively low cost and ease of deployment. However, within gait assessment, one size does not fit all; prescription and multi-modal approaches (e.g., IMU assisted by computer vision) may be more appropriate to ensure a more effective assessment and to address the shortcomings in sensing modalities. By harnessing the power of the IoT, sensing could effectively communicate within a gait assessment context. In particular, with the use of a smartphone, assessments could be carried out in a range of environments with data and analysis facilitated by the ubiquity of the cloud. Furthermore, given the prevalence of artificial intelligence in modern approaches (e.g., computer vision), offloading complex processes to cloud computing instances, a wider range of assessments could take place. Placing data within the cloud ensures that the data can be accessed in real time by all, contributing towards pragmatic gait analysis and new opportunities.

## Data Availability

No new data were created or analyzed in this study. Data sharing is not applicable to this article.
